# Supplementary material for: Deep Learning Evaluation of Glaucoma Detection Using Fundus Photographs in Highly Myopic Populations
Source: Biomedicines. 2024 Jun 23;12(7):1394. doi: 10.3390/biomedicines12071394 (PMC11274657; doi:10.3390/biomedicines12071394)
Supplement: Supplementary file 1 [file biomedicines-12-01394-s001.zip › biomedicines-3010837-supplementary.pdf]

Dataset Description

Our dataset comprises retinal images from various classes, each representing different degrees of high myopia and the presence or absence of glaucoma. Below are some example images from the dataset:

- **High Myopia**: **Figure (a),(b)**
- Disc Deformation: With worsening high myopia, the optic disc horizontally stretches into an oval shape in fundus photography.
- Disc Tilt: High myopia may cause sagittal rotation of the optic disc, resulting in disc tilt.
- Neuroretinal Rim Changes: Eyes with high myopic glaucoma exhibit abnormal neuroretinal rim shapes that no longer conform to normal rules.

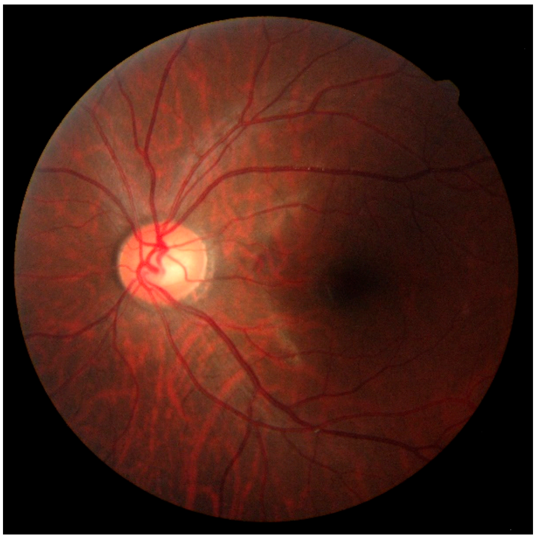

(a)

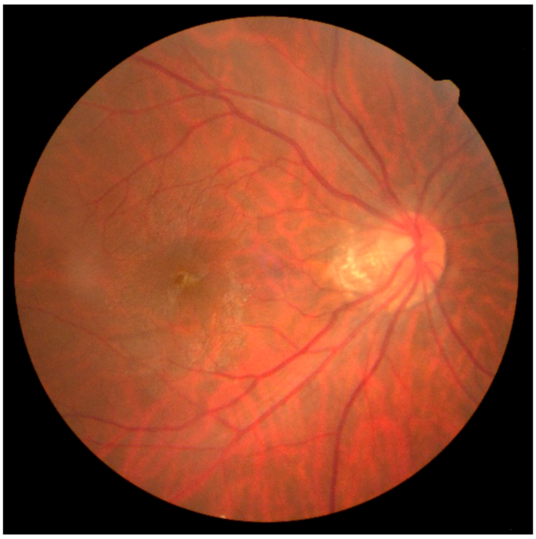

(b)

- **Glaucoma**: **Figure (c),(d)**
- Optic disc cupping: This typically manifests as a reduction in the size of the optic disc, narrowing of its margins, and may even include excavation of the optic disc.
- Neuroretinal Edge Loss: In glaucoma, there is progressive loss of the neuroretinal edge, which typically advances in specific patterns. This loss initially occurs in the inferotemporal region, followed by the supratemporal, infranasal, and supranasal areas as the disease progresses.

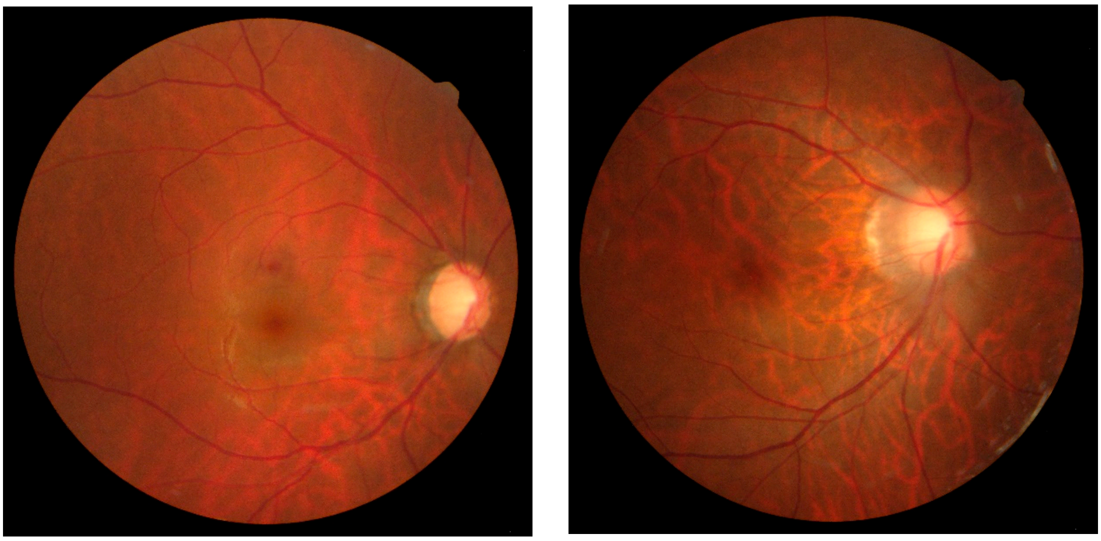

(c) **(d)**

By including these sample images, readers can gain a better understanding of the structure and content of our dataset, as well as the focus and objectives of our study.
